# Teeth with Exposed Pulps

**Published:** 1886-07

**Authors:** B. Merrill Hopkinson

**Affiliations:** Baltimore, Md.


### TEETH WITH EXPOSED PULPS. DEVITALIZATION AND SUBSEQUENT TREATMENT.

BY B. MERRILL HOPKINSON, D. D. S., M. D., BALTIMORE, MD.

I will venture the assertion that the literature of the above subject is more voluminous than that of any other known to our specialty, and it is indeed reasonable that it should be so, for it is a subject of great comprehensiveness. The management of this class of teeth is surrounded by all sorts of difficulties, and with too many in the profession a vast deal of uncertainty, and demands on the part of the operator, in most cases, his highest skill and most painstaking care. It would appear that the subject under consideration is really little understood, notwithstanding the fact that patients daily seek relief from pain due to aching and exposed pulps, and everyone practising our art has ample opportunity to study the treatment of the dental organs in this condition, as well as the opportunity of reading and profiting by the good advice of our eminent men. I am obliged to say that my observation teaches me that the majority of men fail in their treatment. I believe the large number of failures to be due to one of two causes, viz.: either carelessness or improper manipulation. This want of success calls forth the indiscriminate condemnation of pulpless teeth by certain gentlemen in general practice, who, I am sorry to say, do not understand the subject, and draw their conclusions, naturally enough, from those cases they meet with, in which serious trouble has arisen after a pulpless tooth has been filled. I have desired for a long time past to write this article, but owing to numerous other duties I have been obliged to put it off, until, having recently relieved myself of several matters which occupied a great deal of valuable time, I am now able to carry out my intention, and I have the honor to submit the present paper to the profession. I have no desire, for the most part, to lay any claim to originality, nor do I regard myself as infallible, but I must say that after a moderately successful career of over seven years of active practice, with the records of hundreds of pulpless teeth filled, I have yet to learn of the first one which, having been declared satisfactory, has ever returned to me with a history of subsequent annoyance. It may be said that seven years is too short a time to decide whether such teeth may not give rise to serious trouble, and also, that some of my clients for whom I have operated may possibly have failed to return to me, but have sought advice, and perhaps consolation, from some one else about an aching member. With regard to the first proposition, I will say that I regard seven years quite long enough to test a filled pulpless tooth, as well as quite long enough from time of operating for the patient to return to that state of dust from which we are all supposed to have sprung originally, and not require teeth, and if a filled tooth lasts seven years and performs its proper function satisfactorily, all reasonable people will, I am sure, agree in saying that the original operation was well done. I will answer the latter proposition thus: I am not aware of losing any of my clients, still, as I said before, it is possible, and I can only say if any of my operations have been unsuccessful I am not aware of it, but would be most happy if there are such cases, if I could have them brought to my notice. Let us begin at the beginning, and for convenience of description I will take a case from practice.

Enter an individual in distress, either desiring extraction or relief from pain by "killing the nerve," as the laity are pleased to express it. I find a second molar in the left superior maxilla very carious upon the distal, extending to and including a small part of the grinding surface. After carefully cleansing the cavity with a warm carbolized solution, and making a thorough examination, cutting away more of the grinding surface if necessary, I find an exposed pulp. The patient gives a history of continuous pain for a considerable period, and as I pass a spoon excavator over the exposure, slightly enlarging it, I discover an oozing of pus. The indication to me is to devitalize immediately. A small pellet of absorbent cotton is saturated with pure wood creasote; upon this is placed, with a small hoe excavator kept for the purpose, as much arsenic as can be picked up upon the hoe, say gr. $\frac{1}{40}$, and the pellet is then covered completely with sulphate of morphia. In a majority of cases I use a considerable quantity of morphia, which does not interfere with the intended action of the irritant poison, but in all cases, in my experience, gives immunity from pain; indeed, in many cases the sudden cessation from agonizing pain is marvelous, and I have frequently had the pleasure of seeing a patient fall asleep in my chair after passing days and nights of intense suffering. The double action of morphia is of course generally known, and when applied topically it acts upon the periphery of nerves, as in a case of odontalgia, and upon the nerve centers when administered *per orem*, or by hypodermatic method. The knowledge of the local action causes me to prepare my own devitalizing application each time I require it, so that I may vary the quantity of morphia as required in each individual case, and I regard this as of incalculable benefit in the beginning of treatment of exposed pulps, especially in those cases when the aching has been prolonged and violent. Another equally great advantage in preparing the application each time required, is that the quantity of arsenic can also be varied, and I am sure there is as much need of this variation in different cases as there is in the administration of potent drugs in general practice, in the treatment of different individuals. The quantity of creasote is also a prime factor of success in the reduction of pain. I regard the so-called "nerve paste" as very inferior to individual preparations, and a premium offered by the shops on laziness, as well as an incentive to pursue a miserable routine practice which is always condemnable. I would advise a universal avoidance of all such preparations by practitioners, and at least a trial given to the above method by those who have never practised it. The pellet, as prepared, is carried carefully into the cavity and covered over with a dry pellet; both are loosely impacted, and merely the surface of the latter piece coated over with sandarach varnish, being careful not to crowd the application so as to give rise to pain, and also not to allow any of the varnish to find its way into the cavity where it may act as an irritant. If there be no adjoining tooth, or if necessary for any reason, the application is securely ligated in its place. In my opinion the necessity for a covering of wax or gutta percha does not exist, and I regard their application as useless, unless indeed in using "nerve paste" we think we are using gr. $\frac{1}{40}$, when in truth we are using gr. $\frac{1}{4}$, $\frac{1}{2}$, or more, and there is danger of it being "pasted" over the gums or swallowed; in such a case I would certainly advise hermetical sealing. After a very large experience with this class of teeth I have never known of any gingival irritation following my applications, and, indeed, it is impossible that it should occur. I might add that while making the application I

guard the cavity from moisture by means of a napkin. Such an application I allow to remain as near twenty-four hours as may be convenient. As a rule, I find upon removing it, after the lapse of that time, that it has accomplished the desired purpose and I proceed to remove the *debris*. Before speaking of the manner of opening the pulp cavity and canals I must mention that, for some time past, in the cases I have chosen for the following method of practice, I have had gratifying success, viz.: with the use of hydrochlorate of cocaine, as well as with the Fl. Extr. of Cannabis Indica, in removing the pulps of teeth upon the first visit of the patient. In these cases I apply the dam, make an application, allowing it to remain from five to ten minutes, proceed as far as possible, make a second, perhaps a half dozen, and in this way remove the pulp by degrees; sometimes there may be slight pain, often the operation is painless. This method occupies more time than a man in full practice is usually able to devote to it, and until we find an agent which will anæsthetize the pulp more thoroughly and rapidly, most of us will continue the time-honored and safe method of devitalization with arsenic. I regard a second application of arsenic as an impediment to ultimate success, and think the cases are very rare when it is necessary to use it a second time; still, if necessary, a smaller quantity should be used. Where there is considerable pain experienced in the removal of the *debris* after supposed devitalization, I think it a good plan to employ cocaine or cannabis indica, and in such cases they act much more rapidly. I also find in teeth of very dense structure that the application of zinc chloride, in full strength, to the canals, acts most happily, and enables me to remove the remains easily and painlessly. This pain is due to the membrane lining the canals and pulp cavity; it is continuous with the periosteum, and in some cases has great power of resistance, the devitalizing application seeming to have but little effect upon its vitality.

The first thing requisite to remove a dead pulp from a tooth is to gain access to each root in the direction of its long axis. This is a point upon which I am most decided, and in the case under consideration I chisel and burr away the grinding surface until I can enter each root straight. I have read remarkable stories of operators working through one-eighth inch openings in the grinding surface of a tooth, rolling pulpstones backwards and forwards, removing every particle of dead pnlp, aud filling to the apex through such openings. I emphatically believe that in order to succeed universally, we must have room, and plenty of it, and I firmly believe my success with this class of operations has been due in a large measure to giving myself ample room to treat each case properly. I always regret the necessity which obliges me to sacrifice solid tooth structure, but the necessity being present the sacrifice must follow. In cases of anterior teeth I invariably open from the palatine surface to gain a straight canal, unless the decay is so extensive that I am able to gain it through the cavity already formed by caries. I will say just here that I keep my "nerve broaches," and all such "tricks," securely under lock and key, and only use them when for unavoidable reasons I am unable to enter a canal with a drill. That unfortunate condition has presented itself less than half a dozen times in seven years. In gaining access to the canals the pulp cavity is of course thoroughly opened, and the pulp remains removed, as a rule, with an ordinary bur. The entrance to each canal is then slightly enlarged with a suitable bur, and after finding the direction of the canals with a probe, I remove the pulp from them with flexible burs of various sizes, going as near the apicial foramen as possible, and in so doing use very little force upon the hand piece. In using these canal drills I have never drilled through the side, nor forced one through the apex of a root. Do I always go to the apex? I go as far as possible, not to do any harm, and a sufficient distance to attain success; further than this deponent saith not. Great care is of course required in using these engine points in a root canal, but I am of the opinion that with proper handling they are as harmless and at the same time as valuable instruments as we have at our command in the treatment of devitalized teeth. In removing the pulp from small or flat roots I often enlarge them, but my progress is very slow, and I use my probe constantly to discover the direction of the canal. I frequently find, as I suppose everyone does, that the canals of anterior buccal roots of superior molars are very small, and many times so small that they are not discoverable. Those usually belonging to the flat variety are anterior root canals of inferior molars and an occasional bicuspid root canal. Third molar roots are very uncertain, and I rarely attempt to save these teeth if the pulp cannot be saved alive.

To proceed with my case. I have removed all the pulp, or to be modest, all I could find, and, as is my practice in those cases which I have devitalized, I wind my probe with a thin film of absorbent cotton, dip it into carbolic acid and pack it carefully into the root, repeating the same process in each root, always protecting the tooth from moisture; the main cavity I usually fill with gutta percha. My habit is to allow this filling to remain three days when possible, as a test, and at the end of that time to fill the roots and pulp cavity. I prefer metal for a root filling, either gold or tin foil. I have used gutta percha cylinders successfully when a portion of the end of a root has been absorbed, or a foramen enlarged from disuse; in such canals they can be applied when soft most satisfactorily. I cut the foil, No. 4, and fold it into narrow strips, one-fourth of a sheet at a time; these strips are then divided into very small pieces and carried with a small root plugger to the end of the canal. As an antiseptic precaution I always dip the first piece in carbolic acid, other moisture being excluded by means of napkin or dam. I invariably fill three-fourths of the root in this way, and the remainder, together with a portion of the pulp cavity, with gutta percha, preferring not to continue the metal from the orifice of the cavity of decay to apex of root, but rather have an intervening non-conductor, which I believe to be of service even in a pulpless tooth. With regard to the crown filling but little is necessary to say, as I suppose the main features of my operating are the same as those of most of my brothers. Suffice it to say that large complicated cavities in posterior teeth are not filled by me with gold, but with a plastic filling always, and I usually defer this operation for three days from the date of filling roots and pulp cavity.

I have said that I have yet to learn of a single case of a filled pulpless tooth which has returned to me, having given subsequent trouble, which I had declared satisfactory at the time I filled it. There have been cases, some of which I can now recall, that were most unsatisfactory to me, and at such time the patient has been apprised of the fact, and told it was possible that at some future time he might be inconvenienced by the tooth again giving trouble, and warned to return upon the first approach of pain or soreness. Some have returned, and the trouble has been invariably aborted if the patient was seen early enough; if not, by appropriate treatment the more advanced periosteal irritation has been removed, and I have no record of severe periostitis or alveolar abscess. I have often refused to treat certain teeth, rather advising extraction, and in this way undoubtedly saved myself the annoyance of failure of success. In this class I would mention third molars, as a rule; teeth with very crooked roots plainly discoverable, and certain cases when it has been a physical impossibility to gain straight, or anything like straight, access to the root canals, although certain conditions have been present which have caused me to try to save even such teeth. I have said nothing of my treatment of teeth when the pulps have died without the aid of the specialist, and I will briefly say that this class, provided there be not an abscess or periostitis to complicate them, are most easily treated, and we have many and valuable agents at our command to combat successfully all pathological conditions. The treatment of alveolar abscess or periostitis does not properly come under the title of my paper, but they are without doubt frequent complications; my desire is merely to speak of my mode of devitalizing, preparing and filling the root canals and pulp cavity of such teeth as require devitalization. I will say, in conclusion, that I do not claim in this paper to be able to accomplish that which any other operator may not also accomplish, but I am fully convinced that by the mode of operating, as described, the chances of success are more fully assured than by the uncertain modes as practiced by, at least so far as I know, the majority of operators.

