# Corrigendum

**Published:** 1886-08

**Authors:** 


### CORRIGENDUM.

In the article entitled "Teeth with Exposed Pulps," page 355, line 12, in the July No. of the PRACTITIONER, please read "or a foramen enlarged from *disease;*" for, "a foramen enlarged from *disuse.*"

B. MERRILL HOPKINSON, D. D. S , M. D , Baltimore, Md.

